# Elviz – exploration of metagenome assemblies with an interactive visualization tool

**DOI:** 10.1186/s12859-015-0566-4

**Published:** 2015-04-28

**Authors:** Michael Cantor, Henrik Nordberg, Tatyana Smirnova, Matthias Hess, Susannah Tringe, Inna Dubchak

**Affiliations:** Department of Energy, Joint Genome Institute, 2800 Mitchell Drive, Walnut Creek, CA 94598 USA; University of California at Davis, One Shields Avenue, Davis, CA 95616-8521 USA

## Abstract

**Background:**

Metagenomics, the sequencing of DNA collected from an entire microbial community, enables the study of natural microbial consortia in their native habitats. Metagenomics studies produce huge volumes of data, including both the sequences themselves and metadata describing their abundance, assembly, predicted functional characteristics and environmental parameters. The ability to explore these data visually is critically important to meaningful biological interpretation. Current genomics applications cannot effectively integrate sequence data, assembly metadata, and annotation to support both genome and community-level inquiry.

**Results:**

Elviz (Environmental Laboratory Visualization) is an interactive web-based tool for the visual exploration of assembled metagenomes and their complex metadata. Elviz allows scientists to navigate metagenome assemblies across multiple dimensions and scales, plotting parameters such as GC content, relative abundance, phylogenetic affiliation and assembled contig length. Furthermore Elviz enables interactive exploration using real-time plot navigation, search, filters, axis selection, and the ability to drill from a whole-community profile down to individual gene annotations. Thus scientists engage in a rapid feedback loop of visual pattern identification, hypothesis generation, and hypothesis testing.

**Conclusions:**

Compared to the current alternative of generating a succession of static figures, Elviz can greatly accelerate the speed of metagenome analysis. Elviz can be used to explore both user-submitted datasets and numerous metagenome studies publicly available at the Joint Genome Institute (JGI). Elviz is freely available at http://genome.jgi.doe.gov/viz and runs on most current web-browsers.

**Electronic supplementary material:**

The online version of this article (doi:10.1186/s12859-015-0566-4) contains supplementary material, which is available to authorized users.

## Background

Metagenomics is the study of DNA sequence data from genomes of the microbial community (the metagenome) associated with an environmental sample. These data can provide previously unattainable insights into the types of microorganisms that make up a system and the processes that they mediate. Metagenome sequencing has changed our understanding of energy metabolism in the oceans [1], biomass degradation in the gut of termites and cows [2,3], microbial bioremediation of metals and hydrocarbons [4,5], and the human microbiome [6], but its power has been limited by the difficulty to explore microbial communities at multiple levels of granularity to gain a systems-level understanding of their role in the habitat.

Metagenome datasets are produced by shotgun sequencing [7] after which individual sequence reads are usually assembled, annotated, and assigned to individual organisms. De novo assembly of sequence data provides improved accuracy of sequences by removing most random sequencing errors and results in longer and more specific contigs than found in unassembled sequencing reads [8]. Assembly – the process of combining sequence reads into contiguous stretches of DNA (“contigs”) – is based on sequence overlap between reads [9]. The consensus sequence for each contig is either based on the highest quality nucleotide in the aligned reads at each position or based on majority rule. The number of reads underlying each consensus base is called “depth” or “coverage”. Coverage serves as an indicator of both the quality of the assembled sequence and the abundance of the organism from which it derives. These assemblies are extremely challenging to analyze because they contain sequences from many organisms at different abundance levels, usually combined into a single file that may contain hundreds of thousands of contigs, which lack meaningful context. Phylogenetic prediction and annotation usually follow the assembly process: each contig is scanned for known DNA features (genes, protein domains, etc.) which are assigned to putative functions and taxa using homology based prediction methods (for instance, see [10]).

Visual analysis of metagenome data at the level of gene, genome, and ecosystem is of critical importance due to the huge volumes and complexity of the data produced, yet relatively few interactive visualization tools are specifically geared toward microbial community data. Several visualization tools or packages exist for microbial community data, but most (e.g., Mothur [11] and QIIME ([12,13]) focus on phylogenetic profiling using 16S rRNA or other marker genes. Others visualize coverage data integrated with alignment information (e.g., MGAviewer [14]) or comparative analysis of complex metagenome data (e.g., Megan [15], MG-RAST [16] IMG/M-ER [10]), but produce mostly static images. These tools, as well as current non-visual metagenome analysis platforms, treat metagenomes essentially as low quality genome and annotation data. Adding consideration of the rich information on organism abundance and adaptation conveyed by contig (and gene) sequence depth and heterogeneity [10,17-20] offers multiple advantages. To explore the relationships among these metadata (e.g., to investigate coverage vs. phylogenetic prediction in a sample) investigators must still create a static plot, which, using conventional methods, requires time-consuming manual steps. Changing display parameters or exploring different relationships within the data requires repeating these steps. This process is slow, and it requires that the investigator knows exact questions to ask beforehand.

To facilitate comparative metagenomics analyses, we designed a web-based interactive tool, Elviz, that eliminates time-consuming manual step in the analysis of metagenome assemblies. Elviz enables the interpretation and visual exploration of assembled metagenome data, including sequence composition, assembly metrics, preliminary functional predictions, and phylogenetic affiliations. Integration of this information can aid in quickly defining microbial community structure and retrieving sequences and annotations of specific subsets of the data. These capabilities create a true discovery tool that allows for the recognition of phenomena before they can be quantified. Similar recognition tools have been revolutionary for other data-intensive fields (see http://www.nsf.gov/news/special_reports/scivis/winners_2012.jsp) and while Elviz has been developed to address questions predominately relevant to microbiologists and specifically to provide the infrastructure necessary to explore metagenome datasets, most of the framework, libraries, and user interface of Elviz can also be utilized for visualizing data from areas other than microbiology.

## Implementation

### Elviz architecture

Elviz is a web application, written primarily in AngularJS, JavaScript, and WebGL, and nearly all of the logic and computation occurs on the “client” side, in the browser. Users can load their own data into Elviz or explore metagenome assemblies created at the Joint Genome Institute (JGI) and provided through the “server” side of Elviz, a thin REST server, written in Java, that sends data to the client in JSON or tabular text format.

Web browser vendors have put great effort into making the web platform a viable environment for fully featured applications that previously were squarely in the domain of the desktop operating system. This creates the opportunity to develop tools that harness the benefits of the internet (e.g., platform-independence, no need to install or setup software, connectivity to other resources, and the ability to share views with other users) while preserving the computational power and graphical interactive interfaces that were previously limited to the desktop environment.

Elviz takes advantage of two recent technological developments that have greatly accelerated the ability to create rich, efficient, and interactive visual tools on the web, namely WebGL (http://www.khronos.org/webgl/) and HTML5, in particular the LocalStorage API (http://www.w3.org/html/wg/; http://www.w3.org/TR/webstorage/).

WebGL is a web-based implementation of the GL framework, which allows a web application to execute graphics commands using the client computer’s Graphical Processing Unit (GPU). The GPU is specialized hardware for graphics processing, with most graphics cards now supporting hardware-accelerated 3D rendering. Elviz uses this capability to increase the number of objects (i.e., metagenomic contigs) that can be displayed and manipulated in a responsive fashion. Elviz also leverages the wide variety of advanced visual effects available in the WebGL API to differentiate selections and show varying data parameters. The LocalStorage API provides access by the browser to the native file system. With the ability to store and access files on the user’s computer, an application can minimize expensive transfers of data between the server and the client. Additionally, this enables the local exploration of private datasets. LocalStorage also offers the possibility of caching remote datasets so that the user can revisit work in progress without retrieving information from the server.

Elviz has been successfully tested with Google Chrome, Mozilla Firefox, and Opera on OS/X and Windows, with Internet Explorer 9 on Windows, and with Safari on OS/X.

### The Elviz graphical interface

**Figure 1:**
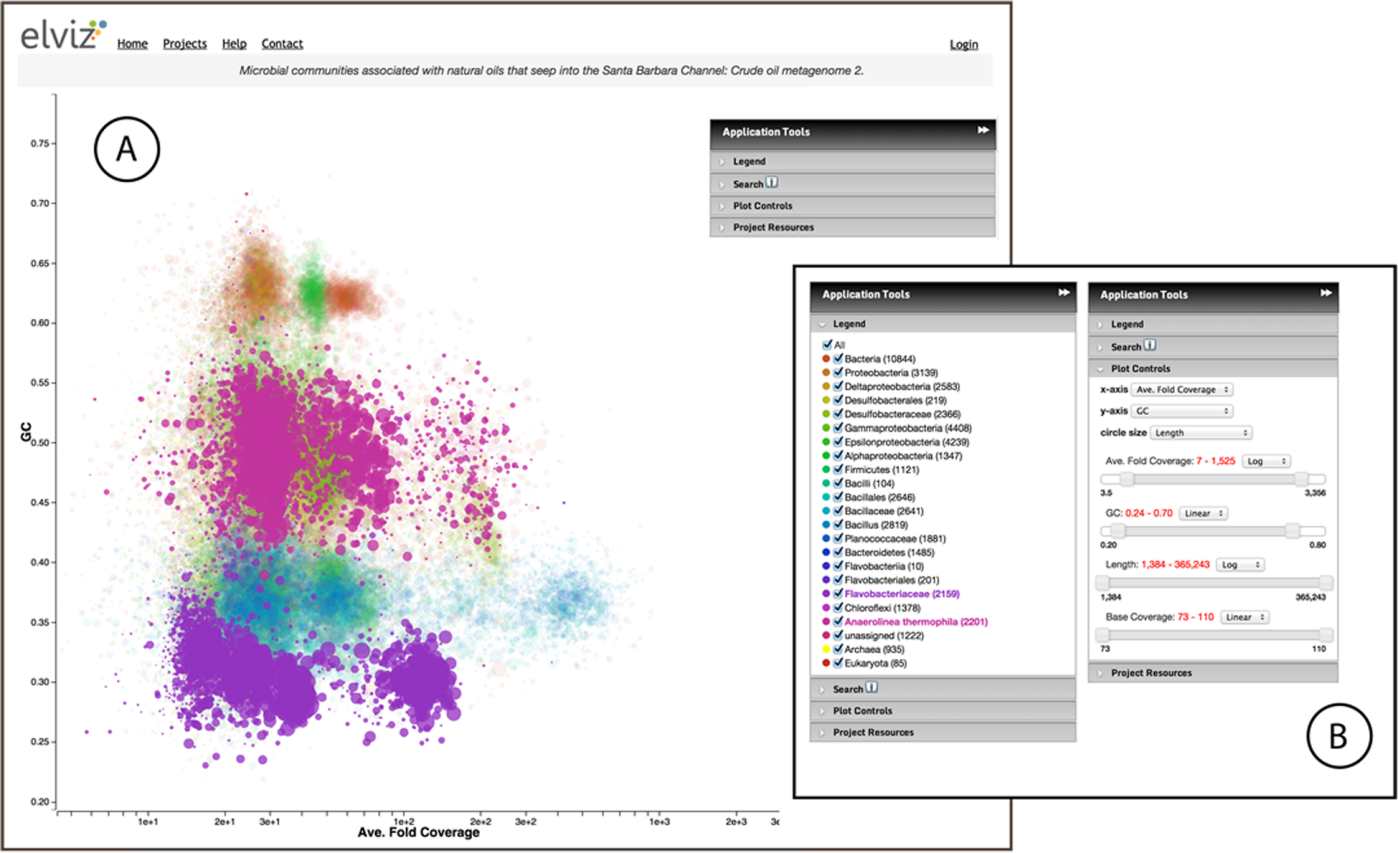
The Elviz interface. **A**. The bubble-plot displays assembled contigs. The floating “Application Tools” panel in the upper right controls plotting parameters. **B**. “Application Tools” are shown with two different panels expanded. The Legend Panel (Figure 1B left) controls the coloring and highlighting of different data groups in the plot. Here, contigs predicted to belong to *Flavobacteriaceae* and *Anaerolinea thermophilia* are highlighted. The Plot Controls panel (Figure 1B right) controls axis selection, plot navigation, and data filtering.

**Figure 2:**
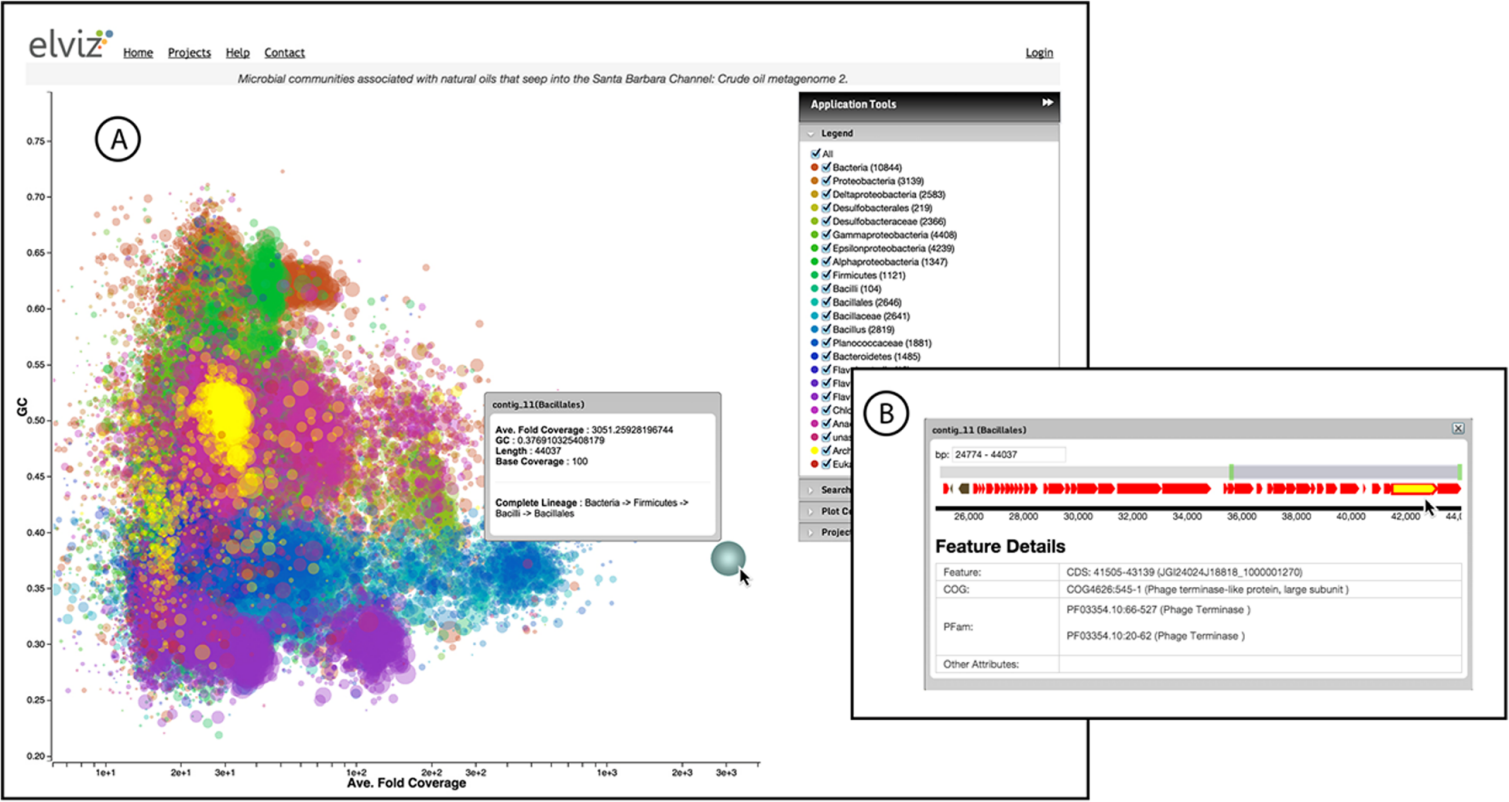
Exploring individual contigs. **A**. When the mouse moves over contig_11 in the plot, a panel appears showing all of the metadata for this contig. **B**. Clicking on the contig brings up the Contig Detail Viewer. Here the user can explore gene annotations on the contig, navigating with the slider at the top of the panel. Red and black glyphs represent predicted gene models in forward and reverse orientation respectively. The yellow-filled gene model is currently selected. Details for this annotation appear in tabular form, including the feature name and position, and predicted COG and Pfam classifications.

The Elviz interface is comprised of two primary components: (1) an interactive bubble-plot displaying metagenomic contigs (Figure 1A), and (2) a floating panel of controls (“Application Tools”) for configuring and manipulating the plot (Figure 1B).

#### The plot and the legend panel

Each point in the Elviz plot represents a single contig of the assembled metagenome. Points are displayed along four user-controllable dimensions: x-axis, y-axis, point size, and point color. For instance, in Figure 1A, GC content is plotted on the y-axis while “Ave. Fold Coverage” – a measure of contig abundance – is plotted on the x-axis. The color of each point indicates the predicted taxonomic assignment of the contig (shown in the Legend Panel of the Application Tools; Figure 1B) while the point size is proportional to the length of each contig. The particular choice of parameters in this example is designed to support a visual assessment of the quality of taxonomic assignments in the sample. Contigs that derive from genomes of the same organism should have similar GC content and read coverage as compared to contigs that derive from separate organisms. Thus, if contigs of a particular color cluster together over these axes (GC content and coverage), the corresponding taxonomic assignment is corroborated.

The Legend panel allows the user to hide, highlight (brighten), and specify colors for contigs assigned to specific phylogenetic groups. These groups can be specified in a variety of ways, as will be described later. In addition, hovering the mouse over legend entries temporarily highlights the corresponding points in the plot, allowing for quick identification.

With JGI metagenomes, such as the example shown in Figure 1, colors are assigned for a finite set of phylogenetic classifications. This set is determined by an algorithm run during preparation of the datasets that determines the taxonomically “deepest” set of 25–30 taxa that can account for all of the contigs. The complete phylogenetic classification for each contig is, however, preserved in a metadata field called “Complete lineage” which can be seen when hovering the mouse over a contig (Figure 2A). This field, like all other contig metadata, is scanned when using Search (see below), making it possible to locate the set of contigs belonging to a taxon at any phylogenetic level.

#### Plot interaction and navigation

The ability to distinguish and visually separate points or groups of points within a dense and overlapping plot represents a central challenge in the visualization of large datasets. Elviz provides a number of features, including plot navigation, filtering, and search, that help to identify contigs of interest.

The Elviz plot can be navigated with mouse operations as well as through the “Plot Controls” panel (Figure 1B). Zooming and panning are accomplished by using the mouse scroll wheel and by click-dragging within the plot, respectively. The same result can be accomplished by setting the boundaries of the axes using their respective sliders. Sliders for variables other than the x- and y-axis operate as filters, which allow the user to reduce visual noise (e.g., filtering out smaller contigs) in the plot or to focus only on contigs within specific parameter ranges.

Within the plot, hovering over any contig brightens this point and displays a panel showing the details of the given contig. When using Elviz with JGI projects, clicking on a point opens the “Contig Detail Viewer” in which the user can navigate along the contig to explore predicted genes and other functional annotations (Figure 2).

**Figure 3:**
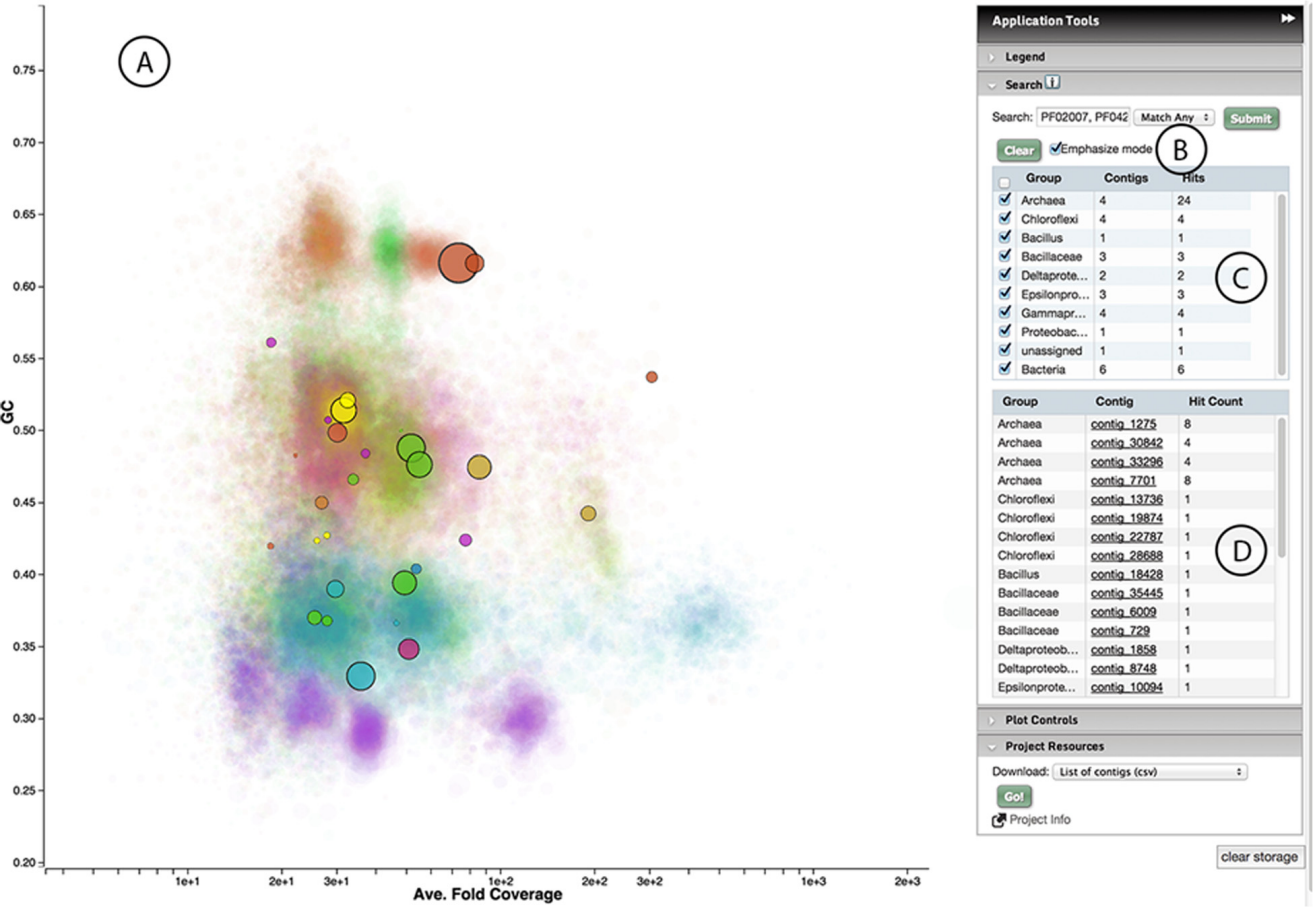
Elviz search. **A**. Contigs containing one or more of eight PFAM domains from the *mtr* gene (see text, Section 2.3) are identified using search, appearing as solid circles ringed in black. Point color still represents predicted phylogeny (see Figure 1B, left) creating a ready visual indication of the distribution of search results across taxa in the sample. **B**. Because “Emphasize mode” is checked, contigs containing search terms are emphasized and others de-emphasized. **C**. **D**. Search result tables present the distribution of contigs with hits across taxa and a count of hits within each contig.

Contigs on the Elviz plot are searchable. Figure 3 shows the results of using the “Search Controls” to locate contigs containing a particular Pfam annotation. Matching contigs appear as black-outlined circles in the plot and are presented in two tables. The first table shows hit counts for each “group”, which can be shown or hidden using the associated check boxes. The second table lists the individual contigs. When viewing JGI projects, clicking on these contigs will bring up the Contig Detail Viewer.

Via the “Download Panel”, Elviz supports the export of subsets of contigs from search or visual selection (enabling or disabling groups or group search results) in a variety of formats, including CSV, and, when available for JGI datasets, GFF (annotations) and FASTA (contig sequences).

### Case study: metagenomics of the Santa Barbara channel oil seep

Here we demonstrate the use of Elviz to investigate the metagenome of the microbial community associated with natural crude oil that seeped into the Santa Barbara Channel [21]. The assembled metagenome contains a total of 803,203 contigs (representing 495,862,225 bp) of which 91,522 are at least 1 kb long and only 6 exceed 50 kb in length. This dataset is publicly available for exploration with Elviz at: http://genome.jgi.doe.gov/viz/plot?jgiProjectId=1019848. A download link for this dataset is also provided at the beginning of the “Upload” section of help (http://genome.jgi.doe.gov/viz/help) in order for users to try out the various features of custom data upload.

Looking at the plotted GC vs. read coverage for this dataset, the users’ eyes are immediately drawn to the putative *Bacillales* point, contig_11 (Figure 2A), which is both very large (44Kb) and noticeably over-represented in the sample (average fold coverage = 3051). Clicking on this contig brings up the “Contig Detail Viewer” (Figure 2B), allowing us to explore predicted gene models and functional annotations in the sequence. Contig_11 contains a large fraction of CDS encoding hypothetical proteins and it is also flanked by CDS that are predicted to encode phage proteins (Figure 2B and Additional file 1: Table S1). It is possible that this contig represents a mobile genetic element composed of individual modules derived from various microbial sources, which might explain the unusual average fold coverage. A more detailed analysis of this contig and its genetic content would be essential before a final conclusion of the contig’s origin and its potential viral nature could be established. Using the export feature we could obtain this contig’s sequence and examine it in further detail.

Prior to examining this assembly with Elviz, we identified the presence of key genes for anaerobic methane oxidation (AMO) within this community, a form of reverse methanogenesis that is energetically favorable when coupled to sulfate reduction. We hypothesize that this microbial activity is of importance for methane (CH_4_) biofiltration during which significant amounts of the CH_4_ released from the seafloor are converted into the less potent greenhouse gas CO_2_ [21]. By analyzing the same data with Elviz one is able to quickly assess the phylogenetic distribution of these genes across the sample and to explore the gene content of each of the contigs containing them. We begin by searching the assembly for contigs that contain Pfam domains associated with AMO (as well as methanogenesis) and conserved in enzymes encoded by *fmd, ftr, mch, mtd, frh, mtr* and *mcr* (Figure 3, showing the search for *mtr* domains). Entries for *mer* and *hdr* were not available from the Pfam database (August 29^th^ 2014) and were therefore not included in our analysis. From the assembled 803,203 contigs, we identify a total of 69 contigs that contained one and 14 contigs that contained two or more conserved regions associated with one of the seven AMO/methanogenesis key enzymes. Twelve of the contigs with two or more AMO/methanogenesis key enzymes were assigned to the *Euryarchaeota*, the phylum that contains the only two phylogenetic groups (ANME-1 and ANME-2) reported to be capable of AMO [22,23]. While these enzymes could also potentially mediate methanogenesis, the homologs in this particular community are most closely related to those in ANME genomes (data not shown). Using the download function in the Project Resources panel (not shown) we exported a CSV file of the contigs identified in the search (along with all of their assembly metadata) for further analysis. The archaeal contigs with AMO/methanogenesis key enzymes can be assigned to six distinct genome bins using their Average Fold Coverage and GC content. Additional file 1: Table S2 summarizes the properties of the contigs that contain a Pfam domain associated with AMO/methanogenesis and that were identified during this study. Contig bins and their assigned contigs are listed in Additional file 1: Table S3. Using Elviz, these results were generated within a short period of time and may now provide the basis for more detailed analyses of the genomic repertoire of this metagenome and some of larger genome bins within it.

### Elviz data

A number of metagenomes produced by DOE’s JGI and annotated in the Integrated Microbial Genomes with Microbiome Samples (IMG/M) database [24] are currently accessible for exploration in Elviz. These projects can be browsed via the “Projects” link at the top of the application.

In addition, users can import their own metagenomic assemblies and annotation into Elviz in an easy and highly customizable fashion using the Elviz upload wizard. The user simply provides a tab or comma delimited metadata file in which each row represents a contig and each column defines a feature (e.g., length or GC content) of the contigs. Column headings must be located in the first row of the table. After uploading the file, the Elviz upload wizard guides the user through a process of assigning columns in the data to the contig id, and default x-axis, y-axis, and point size properties of the plot (these can be changed dynamically once the data is loaded). In this step the user also specifies which columns should be included in the upload. Elviz will automatically assign numerical columns not assigned to plot properties, as “filter” parameters, for which a filter slider will be created. All parameters marked as included (numerical or descriptive) will be displayed in contig popups.

Next, the wizard asks the user to name the column to be used for point color and the method by which the color should be assigned. In the simplest case, the selected column contains ordinal names (e.g., phylogenetic assignments) to which colors can be assigned. In the case of columns containing quantitative values, Elviz supports (1) statistical binning of these values, with a single color then assigned to each bin or (2) creation of a “heat map” such that each point in the plot will be colored along a gradient representing the range of values in the chosen column.

Finally, the wizard provides the user with the option to load annotation data corresponding to the contigs in their dataset. Annotation files are accepted in GFF format.

The user is also given the option to store uploaded data securely and privately on JGI’s Elviz server, provided that a login with the JGI is created. This will allow the user to revisit the imported project later from any computer without having to repeat the upload.

## Results and discussion

Data-intensive fields are often limited by the ability to extract meaning from large datasets. Easily maneuverable software to process and visualize biological (e.g., metagenome) data is critical to leveraging biological meaning from the “omics” datasets now generated in large amounts by thousands of individual investigators around the world. Elviz is a general-purpose tool for visualization of multidimensional data with a set of features that make it of particular value for a visual and efficient exploration of metagenomic data. In addition, Elviz allows one to explore and mine private as well as already published databases. In the example illustrated above, a simple assessment of a metagenomic data set comprised of 803,203 contigs totaling ~495 Mbp generated an instant picture of the distribution of function and phylogeny across the sample, and the immediate visual identification of outliers. This exercise required no bioinformatics expertise or software configuration beyond using a computer with a web browser. With the ability to search and export data from the tool, it was possible to further investigate hypotheses generated from visual exploration with statistical means.

The interface and backend of Elviz are a platform from which a wide range of exploration capabilities will be added in the future. Many metagenomics research projects involve the collection of multiple samples, either from different environments or as part of time series surveys in order to understand dynamics of microbial communities in natural and laboratory conditions. Hence we have identified comparative metagenomics as the most critical next direction for Elviz, and we are currently looking into ways to efficiently visualize the similarities and differences among two or more metagenomic assemblies. Additionally, we recognize a need to integrate metagenomics data with complementary “omics” datasets (e.g., metatranscriptomics and metaproteomics). We are thus exploring methods for visually overlaying these modalities onto assembly data.

## Conclusions

The versatility of Elviz will facilitate metagenomic analyses that would otherwise require extensive bioinformatic skills and a substantial infrastructure, both not readily available to many individual Principal Investigators. Elviz thus represents a valuable contribution to the scientific community, in particular to the field of microbiology and microbial ecology.

## Availability, requirements, and performance

Elviz is freely available at http://genome.jgi.doe.gov/viz. Elviz requires a computer containing a Graphical Processing Unit (GPU) compatible with WebGL rendering in the browser (see http://get.webgl.org/) and runs in web browsers that support WebGL, including Chrome v31+, Firefox v35+, and IE v.11+ (see “http://caniuse.com/#feat=webgl”). Safari, which currently has only partial support for WebGL, is not recommended for use with Elviz. We have successfully tested Elviz using Chrome, Firefox, and Opera in Mac OS/X, Internet Explorer in Windows, and Firefox and Chrome in Linux operating systems. With fairly large datasets (50-100 K contigs), we find that the initial load of the Elviz plot takes from 3–10 seconds over a DSL or Broadband connection (2–15 Mbs download speed). Subsequent loads of the same project take 2 seconds or less when the project has successfully been cached on the client using localStorage.

## Additional file

Additional file 1: Table S1. Genomic features of Contig_11. **Table S2.** Features of contigs containing key enzymes for AMO/methanogenesis. **Table S3.** Archaeal genome bins involved in methane oxidation/methanogenesis.
