# Supplementary material for: Elviz – exploration of metagenome assemblies with an interactive visualization tool
Source: BMC Bioinformatics. 2015 Apr 28;16(1):130. doi: 10.1186/s12859-015-0566-4 (PMC4432942; doi:10.1186/s12859-015-0566-4)
Supplement: Additional file 1: Table S1. — Genomic features of Contig_11. Table S2. Features of contigs containing key enzymes for AMO/methanogenesis. Table S3. Archaeal genome bins involved in methane oxidation/methanogenesis. [file 12859_2015_566_MOESM1_ESM.docx]

Additional file 1

Table S1. Genomic features of Contig_11

| **Start Coordinates** | **End Coordinates** | **Strand** | **Gene Product Name** |
| --- | --- | --- | --- |
| 3 | 1022 | + | Phage terminase-like protein, large subunit |
| 1044 | 2180 | + | Phage-related protein |
| 2183 | 2920 | + | Protease subunit of ATP-dependent Clp proteases |
| 2942 | 4084 | + | Phage capsid family |
| 4105 | 4311 | + | hypothetical protein |
| 4318 | 4623 | + | hypothetical protein |
| 4610 | 4972 | + | Bacteriophage head-tail adaptor |
| 4959 | 5411 | + | hypothetical protein |
| 5408 | 5785 | + | Protein of unknown function (DUF3168) |
| 5801 | 6397 | + | hypothetical protein |
| 6456 | 6800 | + | hypothetical protein |
| 6821 | 7117 | + | hypothetical protein |
| 7104 | 8927 | + | hypothetical protein |
| 8927 | 9754 | + | Phage-related protein |
| 9777 | 11603 | + | hypothetical protein |
| 11626 | 14877 | + | hypothetical protein |
| 14980 | 16821 | + | Protein of unknown function (DUF1142) |
| 16933 | 17217 | + | hypothetical protein |
| 17289 | 17687 | + | hypothetical protein |
| 17702 | 17953 | + | hypothetical protein |
| 17954 | 18955 | + | Putative peptidoglycan-binding domain-containing protein |
| 19086 | 19343 | + | hypothetical protein |
| 19376 | 20188 | + | hypothetical protein |
| 20374 | 20484 | + | hypothetical protein |
| 20489 | 21790 | + | DNA segregation ATPase FtsK/SpoIIIE and related proteins |
| 21729 | 22331 | + | hypothetical protein |
| 22346 | 22873 | + | hypothetical protein |
| 22963 | 23361 | + | YopX protein |
| 23358 | 23516 | + | hypothetical protein |
| 23516 | 23692 | + | hypothetical protein |
| 23801 | 24265 | + | hypothetical protein |
| 25066 | 25275 | + | hypothetical protein |
| 25309 | 25389 | - | hypothetical protein |
| 25612 | 26007 | - | Predicted transcription factor, homolog of eukaryotic MBF1 |
| 26154 | 26363 | + | Predicted transcriptional regulators - COG1476 |
| 26366 | 26476 | + | hypothetical protein |
| 26480 | 26596 | + | hypothetical protein |
| 26633 | 26902 | + | hypothetical protein |
| 26934 | 27155 | + | hypothetical protein |
| 27158 | 27328 | + | hypothetical protein |
| 27325 | 27531 | + | hypothetical protein |
| 27515 | 27676 | + | hypothetical protein |
| 27669 | 27827 | + | hypothetical protein |
| 27830 | 27991 | + | hypothetical protein |
| 28012 | 28224 | + | hypothetical protein |
| 28241 | 28519 | + | hypothetical protein |
| 28731 | 28910 | + | hypothetical protein |
| 28907 | 29515 | + | hypothetical protein |
| 29530 | 29718 | + | hypothetical protein |
| 29734 | 29964 | + | hypothetical protein |
| 29961 | 30731 | + | Protein of unknown function (DUF1351) |
| 30733 | 31359 | + | hypothetical protein |
| 31430 | 33070 | + | DNA polymerase elongation subunit (family B) |
| 33075 | 34877 | + | hypothetical protein |
| 35325 | 35480 | + | hypothetical protein |
| 35477 | 35731 | + | hypothetical protein |
| 35728 | 36435 | + | DNA modification methylase |
| 36540 | 36752 | + | hypothetical protein |
| 36933 | 37148 | + | hypothetical protein |
| 37145 | 37630 | + | hypothetical protein |
| 37627 | 37986 | + | hypothetical protein |
| 37987 | 38544 | + | Uncharacterized protein conserved in bacteria |
| 38534 | 38698 | + | hypothetical protein |
| 38745 | 39002 | + | hypothetical protein |
| 39080 | 39496 | + | hypothetical protein |
| 39600 | 40301 | + | Uncharacterized protein conserved in bacteria |
| 40445 | 40537 | + | hypothetical protein |
| 40769 | 41095 | + | Restriction endonuclease |
| 41200 | 41508 | + | Phage terminase, small subunit |
| 41505 | 43139 | + | Phage terminase-like protein, large subunit |
| 43161 | 44036 | + | Phage-related protein |

Table S2. Features of contigs containing key enzymes for AMO/methanogenesis

| **Gene** | **PFAM ID** | **Contig ID** | **Average coverage** [fold] | **Length** [bp] | **GC**  [%] | **Phylogenetic classification** |
| --- | --- | --- | --- | --- | --- | --- |
| *fmd* | PF02663 | contig_1069 | 30 | 11,095 | 47 | *Bacteria\|Chloroflexi\|Anaerolineae\|Anaerolineales\|Anaerolineaceae\|Anaerolinea\|thermophila* |
| *fmd* | PF02663 | contig_1491 | 25 | 9,614 | 47 | *Bacteria\|Chloroflexi\|Anaerolineae\|Anaerolineales\|Anaerolineaceae\|Anaerolinea\|thermophila* |
| *fmd* | PF02663 | contig_1845 | 65 | 8,828 | 50 | *Bacteria\|Chloroflexi\|Anaerolineae\|Anaerolineales\|Anaerolineaceae\|Anaerolinea\|thermophila* |
| *fmd* | PF02663 | contig_22193 | 199 | 2,531 | 45 | *Bacteria\|Chloroflexi\|Anaerolineae\|Anaerolineales\|Anaerolineaceae\|Anaerolinea\|thermophila* |
| *fmd* | PF02663 | contig_47708 | 40 | 1,588 | 44 | *Bacteria\|Chloroflexi\|Ktedonobacteria\|Ktedonobacterales\|Ktedonobacteraceae\|Ktedonobacter\|racemifer* |
| *fmd* | PF02663 | contig_22957 | 25 | 2,480 | 34 | *Bacteria\|Firmicutes\|Bacilli\|Bacillales\|Bacillaceae\|Bacillus* |
| *fmd* | PF02663 | contig_20062 | 48 | 2,681 | 37 | *Bacteria\|Firmicutes\|Bacilli\|Bacillales\|Bacillaceae\|Halalkalibacillus\|halophilus* |
| *fmd* | PF02663 | contig_18395 | 27 | 2,815 | 55 | *Bacteria\|Firmicutes\|Clostridia* |
| *fmd* | PF02663 | contig_3465 | 25 | 6,563 | 37 | *Bacteria\|Firmicutes* |
| *fmd* | PF02663 | contig_15299 | 18 | 3,121 | 30 | *Bacteria\|Bacteroidetes\|Flavobacteriia\|Flavobacteriales\|Flavobacteriaceae* |
| *fmd* | PF02663 | contig_13707 | 59 | 3,317 | 49 | *Bacteria* |
| *fmd* | PF02663 | contig_13895 | 28 | 3,291 | 48 | *Bacteria* |
| *fmd* | PF02663 | contig_24181 | 14 | 2,410 | 52 | *Bacteria* |
| *fmd* | PF02663 | contig_2971 | 24 | 7,035 | 54 | *Bacteria* |
| *fmd* | PF02663 | contig_35034 | 54 | 1,937 | 62 | *Bacteria* |
| *fmd* | PF02663 | contig_38689 | 34 | 1,825 | 53 | *Bacteria* |
| *fmd* | PF02663 | contig_39840 | 36 | 1,791 | 36 | *Bacteria* |
| *fmd* | PF02663 | contig_49862 | 15 | 1,542 | 42 | *Bacteria* |
| *fmd* | PF02663 | contig_6273 | 29 | 4,927 | 63 | *Bacteria* |
| *fmd* | PF02663 | contig_7738 | 25 | 4,438 | 51 | *Bacteria* |
| *fmd* | PF02663 | contig_967 | 30 | 11,500 | 63 | *Bacteria* |
| *ftr* | PF01913 | contig_6466 | 30 | 4,853 | 51 | *Archaea\|Euryarchaeota\|Methanomicrobia\|Methanosarcinales\|Methanosarcinaceae* |
| *ftr* | PF01913 | contig_35219 | 27 | 1,930 | 59 | *Bacteria\|Proteobacteria\|Alphaproteobacteria\|Rhizobiales\|Hyphomicrobiaceae\|Hyphomicrobium\|zavarzinii* |
| *ftr* | PF01913 | contig_9246 | 44 | 4,066 | 61 | *Bacteria\|Proteobacteria\|Alphaproteobacteria\|Rhizobiales* |
| *mch* | PF02289 | contig_15374 | 30 | 3,112 | 50 | *Archaea\|Euryarchaeota\|Methanomicrobia\|Methanosarcinales\|Methanosarcinaceae* |
| *mch* | PF02289 | contig_839 | 29 | 12,262 | 51 | *Archaea\|Euryarchaeota\|Methanomicrobia* |
| *mch* | PF02289 | contig_12644 | 39 | 3,466 | 64 | *Bacteria\|Proteobacteria* |
| *mtd* | PF01993 | contig_1089 | 30 | 10,991 | 52 | *Archaea\|Euryarchaeota\|Methanomicrobia\|Methanosarcinales\|Methanosarcinaceae* |
| *mtd* | PF01993 | contig_48430 | 15 | 1,572 | 33 | *Archaea\|Euryarchaeota\|Methanomicrobia\|Methanocellales\|Methanocellaceae\|Methanocella\|conradii* |
| *frh* | PF04422 | contig_1035 | 30 | 11,242 | 52 | *Archaea\|Euryarchaeota* |
| *frh* | PF04422 | contig_1581 | 31 | 9,391 | 52 | *Archaea\|Euryarchaeota\|Methanomicrobia\|Methanosarcinales\|Methanosarcinaceae\|Methanolobus* |
| *frh* | PF04422 | contig_35469 | 18 | 1,921 | 46 | *Archaea\|Euryarchaeota* |
| *frh* | PF04422 | contig_47456 | 21 | 1,593 | 47 | *Archaea\|Euryarchaeota\|Methanomicrobia\|Methanosarcinales\|Methanosarcinaceae* |
| *frh* | PF04422 | contig_6443 | 205 | 4,861 | 49 | *Archaea\|Euryarchaeota* |
| *frh* | PF04422 | contig_7595 | 30 | 4,480 | 51 | *Archaea\|Euryarchaeota\|Methanomicrobia* |
| *frh* | PF04422 | contig_9290 | 194 | 4,056 | 46 | *Bacteria\|Proteobacteria\|Deltaproteobacteria\|Desulfobacterales\|Desulfobacteraceae* |
| *frh* | PF04422 | contig_8256 | 26 | 4,289 | 44 | *Bacteria\|Proteobacteria\|Deltaproteobacteria* |
| *frh* | PF04422 | contig_49401 | 15 | 1,551 | 44 | *unassigned* |
| *frh* | PF04422 | contig_1387 | 39 | 9,909 | 38 | *Bacteria* |
| *frh* | PF04422 | contig_34167 | 21 | 1,966 | 60 | *Bacteria\|Actinobacteria\|Actinobacteria\|Actinomycetales* |
| *frh* | PF04432 | contig_1035 | 30 | 11,242 | 52 | *Archaea\|Euryarchaeota* |
| *frh* | PF04432 | contig_1581 | 31 | 9,391 | 52 | *Archaea\|Euryarchaeota\|Methanomicrobia\|Methanosarcinales\|Methanosarcinaceae\|Methanolobus* |
| *frh* | PF04432 | contig_35469 | 18 | 1,921 | 46 | *Archaea\|Euryarchaeota* |
| *frh* | PF04432 | contig_47456 | 21 | 1,593 | 47 | *Archaea\|Euryarchaeota\|Methanomicrobia\|Methanosarcinales\|Methanosarcinaceae* |
| *frh* | PF04432 | contig_6443 | 205 | 4,861 | 49 | *Archaea\|Euryarchaeota* |
| *frh* | PF04432 | contig_7595 | 30 | 4,480 | 51 | *Archaea\|Euryarchaeota\|Methanomicrobia* |
| *frh* | PF04432 | contig_31665 | 24 | 2,058 | 32 | *Bacteria\|Firmicutes\|Bacilli\|Bacillales* |
| *frh* | PF04432 | contig_11524 | 25 | 3,633 | 56 | *Bacteria\|Firmicutes\|Clostridia\|Thermoanaerobacterales\|Thermoanaerobacteraceae* |
| *frh* | PF04432 | contig_24190 | 26 | 2,410 | 54 | *Bacteria\|Firmicutes\|Clostridia\|Clostridiales* |
| *frh* | PF04432 | contig_12747 | 44 | 3,451 | 45 | *Bacteria\|Proteobacteria\|Deltaproteobacteria\|Desulfobacterales\|Desulfobacteraceae\|Desulfosarcina\|sp.* |
| *frh* | PF04432 | contig_23104 | 29 | 2,472 | 53 | *Bacteria\|Proteobacteria\|Deltaproteobacteria\|Desulfobacterales\|Desulfobacteraceae\|Desulfospira\|joergensenii* |
| *frh* | PF04432 | contig_7912 | 139 | 4,390 | 48 | *Bacteria\|Proteobacteria\|Deltaproteobacteria\|Desulfobacterales\|Desulfobacteraceae\|Desulfospira\|joergensenii* |
| *frh* | PF04432 | contig_830 | 49 | 12,315 | 46 | *Bacteria\|Proteobacteria\|Deltaproteobacteria\|Desulfobacterales\|Desulfobacteraceae* |
| *frh* | PF04432 | contig_34175 | 152 | 1,966 | 45 | *Bacteria\|Proteobacteria\|Deltaproteobacteria* |
| *frh* | PF04432 | contig_8256 | 26 | 4,289 | 44 | *Bacteria\|Proteobacteria\|Deltaproteobacteria* |
| *frh* | PF04432 | contig_1387 | 39 | 9,909 | 38 | *Bacteria* |
| *frh* | PF04432 | contig_34167 | 21 | 1,966 | 60 | *Bacteria\|Actinobacteria\|Actinobacteria\|Actinomycetales* |
| *frh* | PF04432 | contig_42510 | 27 | 1,714 | 66 | *Bacteria* |
| *frh* | PF04432 | contig_5144 | 69 | 5,427 | 50 | *Bacteria* |
| *mtr* | PF02007 | contig_1275 | 32 | 10,248 | 51 | *Archaea\|Euryarchaeota* |
| *mtr* | PF02007 | contig_33296 | 26 | 1,997 | 42 | *Archaea\|Euryarchaeota\|Methanomicrobia\|Methanosarcinales\|Methanosarcinaceae* |
| *mtr* | PF02007 | contig_7701 | 32 | 4,449 | 52 | *Archaea\|Euryarchaeota\|Methanomicrobia\|Methanosarcinales\|Methanosaetaceae\|Methanosaeta\|harundinacea* |
| *mtr* | PF02007 | contig_1858 | 86 | 8,805 | 47 | *Bacteria\|Proteobacteria\|Deltaproteobacteria* |
| *mtr* | PF02007 | contig_1292 | 55 | 10,213 | 48 | *Bacteria\|Proteobacteria\|Gammaproteobacteria* |
| *mtr* | PF02007 | contig_795 | 52 | 12,587 | 49 | *Bacteria\|Proteobacteria\|Gammaproteobacteria* |
| *mtr* | PF04206 | contig_1275 | 32 | 10,248 | 51 | *Archaea\|Euryarchaeota* |
| *mtr* | PF04207 | contig_1275 | 32 | 10,248 | 51 | *Archaea\|Euryarchaeota* |
| *mtr* | PF04207 | contig_30842 | 28 | 2,090 | 43 | *Archaea\|Euryarchaeota\|Methanomicrobia\|Methanosarcinales\|Methanosarcinaceae* |
| *mtr* | PF04207 | contig_7701 | 32 | 4,449 | 52 | *Archaea\|Euryarchaeota\|Methanomicrobia\|Methanosarcinales\|Methanosaetaceae\|Methanosaeta\|harundinacea* |
| *mtr* | PF04208 | contig_1275 | 32 | 10,248 | 51 | *Archaea\|Euryarchaeota* |
| *mtr* | PF04208 | contig_30842 | 28 | 2,090 | 43 | *Archaea\|Euryarchaeota\|Methanomicrobia\|Methanosarcinales\|Methanosarcinaceae* |
| *mtr* | PF04208 | contig_7701 | 32 | 4,449 | 52 | *Archaea\|Euryarchaeota\|Methanomicrobia\|Methanosarcinales\|Methanosaetaceae\|Methanosaeta\|harundinacea* |
| *mtr* | PF04210 | contig_1275 | 32 | 10,248 | 51 | *Archaea\|Euryarchaeota* |
| *mtr* | PF04210 | contig_33296 | 26 | 1,997 | 42 | *Archaea\|Euryarchaeota\|Methanomicrobia\|Methanosarcinales\|Methanosarcinaceae* |
| *mtr* | PF04210 | contig_7701 | 32 | 4,449 | 52 | *Archaea\|Euryarchaeota\|Methanomicrobia\|Methanosarcinales\|Methanosaetaceae\|Methanosaeta\|harundinacea* |
| *mtr* | PF04210 | contig_6009 | 30 | 5,033 | 39 | *Bacteria\|Firmicutes\|Bacilli\|Bacillales\|Bacillaceae\|Lysinibacillus* |
| *mtr* | PF04210 | contig_729 | 36 | 12,967 | 33 | *Bacteria\|Firmicutes\|Bacilli\|Bacillales\|Bacillaceae* |
| *mtr* | PF04210 | contig_1799 | 49 | 8,914 | 39 | *Bacteria\|Proteobacteria\|Epsilonproteobacteria* |
| *mtr* | PF04210 | contig_16691 | 34 | 2,973 | 47 | *Bacteria\|Proteobacteria\|Gammaproteobacteria\|Methylococcales\|Methylococcaceae* |
| *mtr* | PF04210 | contig_12529 | 27 | 3,482 | 45 | *Bacteria\|Proteobacteria* |
| *mtr* | PF04210 | contig_3279 | 51 | 6,710 | 35 | *unassigned* |
| *mtr* | PF04210 | contig_15951 | 304 | 3,049 | 54 | *Bacteria* |
| *mtr* | PF04210 | contig_34 | 73 | 33,445 | 62 | *Bacteria* |
| *mtr* | PF04210 | contig_43614 | 22 | 1,684 | 48 | *Bacteria* |
| *mtr* | PF04211 | contig_1275 | 32 | 10,248 | 51 | *Archaea\|Euryarchaeota* |
| *mtr* | PF04211 | contig_30842 | 28 | 2,090 | 43 | *Archaea\|Euryarchaeota\|Methanomicrobia\|Methanosarcinales\|Methanosarcinaceae* |
| *mtr* | PF04211 | contig_7701 | 32 | 4,449 | 52 | *Archaea\|Euryarchaeota\|Methanomicrobia\|Methanosarcinales\|Methanosaetaceae\|Methanosaeta\|harundinacea* |
| *mtr* | PF04211 | contig_19874 | 18 | 2,694 | 56 | *Bacteria\|Chloroflexi* |
| *mtr* | PF04211 | contig_4651 | 83 | 5,697 | 62 | *Bacteria\|Actinobacteria\|Actinobacteria* |
| *mtr* | PF05440 | contig_1275 | 32 | 10,248 | 51 | *Archaea\|Euryarchaeota* |
| *mtr* | PF05440 | contig_30842 | 28 | 2,090 | 43 | *Archaea\|Euryarchaeota\|Methanomicrobia\|Methanosarcinales\|Methanosarcinaceae* |
| *mtr* | PF05440 | contig_7701 | 32 | 4,449 | 52 | *Archaea\|Euryarchaeota\|Methanomicrobia\|Methanosarcinales\|Methanosaetaceae\|Methanosaeta\|harundinacea* |
| *mtr* | PF05440 | contig_35445 | 46 | 1,922 | 37 | *Bacteria\|Firmicutes\|Bacilli\|Bacillales\|Bacillaceae\|Lysinibacillus\|fusiformis* |
| *mtr* | PF09472 | contig_1275 | 32 | 10,248 | 51 | *Archaea\|Euryarchaeota* |
| *mtr* | PF09472 | contig_33296 | 26 | 1,997 | 42 | *Archaea\|Euryarchaeota\|Methanomicrobia\|Methanosarcinales\|Methanosarcinaceae* |
| *mtr* | PF09472 | contig_7701 | 32 | 4,449 | 52 | *Archaea\|Euryarchaeota\|Methanomicrobia\|Methanosarcinales\|Methanosaetaceae\|Methanosaeta\|harundinacea* |
| *mtr* | PF09472 | contig_13736 | 77 | 3,311 | 42 | *Bacteria\|Chloroflexi\|Chloroflexi\|Chloroflexales\|Oscillochloridaceae\|Oscillochloris\|trichoides* |
| *mtr* | PF09472 | contig_22787 | 37 | 2,491 | 48 | *Bacteria\|Chloroflexi\|Chloroflexi\|Chloroflexales\|Chloroflexaceae\|Chloroflexus* |
| *mtr* | PF09472 | contig_28688 | 28 | 2,180 | 51 | *Bacteria\|Chloroflexi\|Ktedonobacteria\|Ktedonobacterales\|Ktedonobacteraceae\|Ktedonobacter\|racemifer* |
| *mtr* | PF09472 | contig_18428 | 54 | 2,812 | 40 | *Bacteria\|Firmicutes\|Bacilli\|Bacillales\|Bacillaceae\|Bacillus\|sp.* |
| *mtr* | PF09472 | contig_8748 | 191 | 4,174 | 44 | *Bacteria\|Proteobacteria\|Deltaproteobacteria* |
| *mtr* | PF09472 | contig_10094 | 25 | 3,891 | 37 | *Bacteria\|Proteobacteria\|Epsilonproteobacteria\|Campylobacterales* |
| *mtr* | PF09472 | contig_18373 | 28 | 2,816 | 37 | *Bacteria\|Proteobacteria\|Epsilonproteobacteria\|Campylobacterales* |
| *mtr* | PF09472 | contig_47711 | 48 | 1,588 | 50 | *Bacteria\|Proteobacteria\|Gammaproteobacteria* |
| *mtr* | PF09472 | contig_31675 | 18 | 2,058 | 42 | *Bacteria* |
| *mtr* | PF09472 | contig_4427 | 30 | 5,841 | 50 | *Bacteria* |
| *mcr* | PF02240 | contig_2160 | 32 | 8,257 | 50 | *Archaea\|Euryarchaeota\|Methanomicrobia\|Methanosarcinales* |
| *mcr* | PF02241 | contig_2160 | 32 | 8,257 | 50 | *Archaea\|Euryarchaeota\|Methanomicrobia\|Methanosarcinales* |
| *mcr* | PF02249 | contig_2160 | 32 | 8,257 | 50 | *Archaea\|Euryarchaeota\|Methanomicrobia\|Methanosarcinales* |
| *mcr* | PF02505 | contig_2160 | 32 | 8,257 | 50 | *Archaea\|Euryarchaeota\|Methanomicrobia\|Methanosarcinales* |
| *mcr* | PF02745 | contig_2160 | 32 | 8,257 | 50 | *Archaea\|Euryarchaeota\|Methanomicrobia\|Methanosarcinales* |
| *mcr* | PF02783 | none detected | NA | NA | NA | *NA* |
|  |  |  |  |  |  |  |
| *mcr* | PF04609 | contig_1020 | 34 | 11,287 | 51 | *Archaea\|Euryarchaeota\|Methanomicrobia\|Methanosarcinales\|Methanosarcinaceae* |
| *mcr* | PF04609 | contig_2160 | 32 | 8,257 | 50 | *Archaea\|Euryarchaeota\|Methanomicrobia\|Methanosarcinales* |
| *mcr* | PF04609 | contig_448 | 62 | 15,428 | 62 | *Bacteria* |

Table S3. Archaeal genome bins involved in methane oxidation/methanogenesis

|  |  | **Genome Bin 1** | **Genome Bin 2** | | **Genome Bin 3** | | **Genome Bin 4** | | | | | | | | | | **Genome Bin 5** | **Genome Bin 6** |
| --- | --- | --- | --- | --- | --- | --- | --- | --- | --- | --- | --- | --- | --- | --- | --- | --- | --- | --- |
|  | **Contig ID** | 48430 | 35469 | 47456 | 30842 | 33296 | 839 | 1035 | 1089 | 1275 | 1581 | 2160 | 6466 | 7595 | 7701 | 15374 | 1020 | 6443 |
| **Gene** | **PFAM ID** |  |  |  |  |  |  |  |  |  |  |  |  |  |  |  |  |  |
| *fmd* | PF02663 |  |  |  |  |  |  |  |  |  |  |  |  |  |  |  |  |  |
| *ftr* | PF01913 |  |  |  |  |  |  |  |  |  |  |  | x |  |  |  |  |  |
| *mch* | PF02289 |  |  |  |  |  | x |  |  |  |  |  |  |  |  | x |  |  |
| *mtd* | PF01993 | x |  |  |  |  |  |  | x |  |  |  |  |  |  |  |  |  |
| *frh* | PF04422 |  | x | x |  |  |  | x |  |  | x |  |  | x |  |  |  | x |
| *frh* | PF04432 |  | x | x |  |  |  | x |  |  | x |  |  | x |  |  |  | x |
| *mtr* | PF02007 |  |  |  |  | x |  |  |  | x |  |  |  |  | x |  |  |  |
| *mtr* | PF04208 |  |  |  | x |  |  |  |  | x |  |  |  |  | x |  |  |  |
| *mtr* | PF09472 |  |  |  |  | x |  |  |  | x |  |  |  |  | x |  |  |  |
| *mtr* | PF04206 |  |  |  |  |  |  |  |  | x |  |  |  |  |  |  |  |  |
| *mtr* | PF04207 |  |  |  | x |  |  |  |  | x |  |  |  |  | x |  |  |  |
| *mtr* | PF04210 |  |  |  |  | x |  |  |  | x |  |  |  |  | x |  |  |  |
| *mtr* | PF04211 |  |  |  | x |  |  |  |  | x |  |  |  |  | x |  |  |  |
| *mtr* | PF05440 |  |  |  | x |  |  |  |  | x |  |  |  |  | x |  |  |  |
| *mcr* | PF04609 |  |  |  |  |  |  |  |  |  |  | x |  |  |  |  | x |  |
| *mcr* | PF02240 |  |  |  |  |  |  |  |  |  |  | x |  |  |  |  |  |  |
| *mcr* | PF02241 |  |  |  |  |  |  |  |  |  |  | x |  |  |  |  |  |  |
| *mcr* | PF02249 |  |  |  |  |  |  |  |  |  |  | x |  |  |  |  |  |  |
| *mcr* | PF02745 |  |  |  |  |  |  |  |  |  |  | x |  |  |  |  |  |  |
| *mcr* | PF02783 |  |  |  |  |  |  |  |  |  |  | x |  |  |  |  |  |  |
| *mcr* | PF02505 |  |  |  |  |  |  |  |  |  |  | x |  |  |  |  |  |  |
|  | **Average Fold Coverage** | 15 | 18 | 21 | 28 | 26 | 29 | 30 | 30 | 32 | 31 | 32 | 30 | 30 | 32 | 30 | 34 | 205 |
|  | **GC** | 33% | 46% | 47% | 43% | 42% | 51% | 52% | 52% | 51% | 52% | 50% | 51% | 51% | 52% | 50% | 51% | 49% |

| **Genome Bin** | |
| --- | --- |
| 1 | Average Contig Fold Coverage <16; Contig GC <35% |
| 2 | Average Contig Fold Coverage 16-24; Contig GC 45%-48% |
| 3 | Average Contig Fold Coverage 24-28; Contig GC 35%-45% |
| 4 | Average Contig Fold Coverage 28-32; Contig GC 50%-52% |
| 5 | Average Contig Fold Coverage 32-100; GC 50%-52% |
| 6 | Average Contig Fold Coverage >100; Contig GC 48%-50% |
